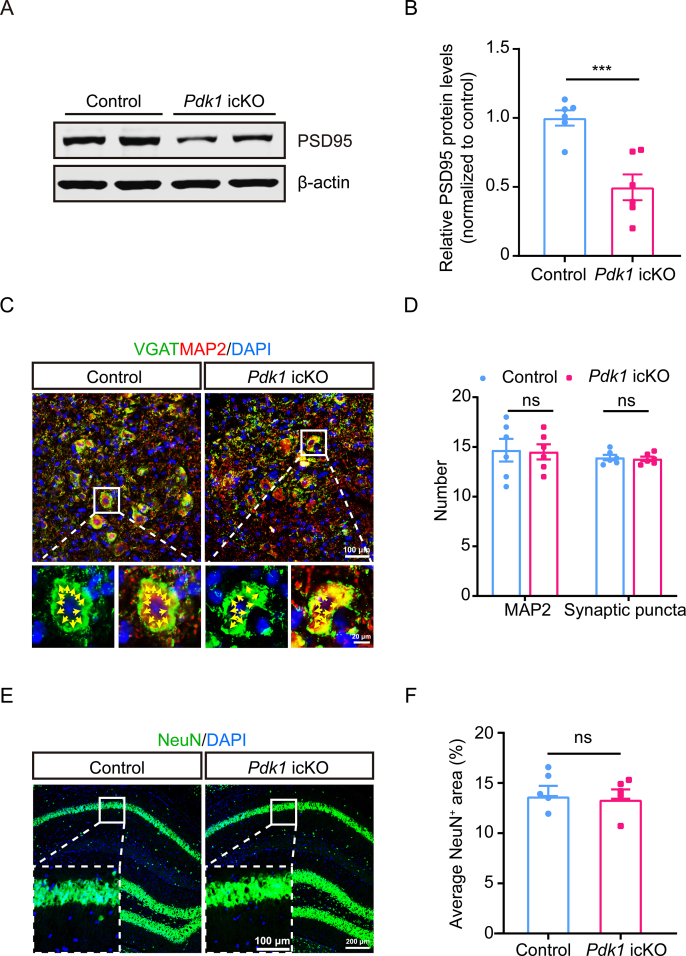# Targeting enhancing myelin regeneration reverses cognitive deficits in a mouse model of intellectual disability

**DOI:** 10.1016/j.neurot.2026.e00906

**Published:** 2026-04-13

**Authors:** Pingping Qiao, He Wang, Pingping Qu, Lifang Guo, Yanbo Zhou, Tingting Zeng, Jiang Chen, Jian Li, Yimin Hu, Guiquan Chen

**Affiliations:** a MOE Key Laboratory of Model Animal for Disease Study, Model Animal Research Center, Suqian Scientific Research Institute of Nanjing University Medical School, Jiangsu Key Laboratory of Molecular Medicine, Medical School, Nanjing University, 12 Xuefu Avenue, Nanjing, 210061, China; b Department of Anesthesiology, Pain and Perioperative Medicine, The First Affiliated Hospital of Zhengzhou University, 1 East Jianshe Road, Zhengzhou, 450052, China; c Jinling Pharmaceutical Company Limited, Jinling Pharmaceutical Building, 238 Zhongyang Road, Nanjing, 210009, China; d Department of Laser Surgery, Hospital of Dermatology, Chinese Academy of Medical Sciences and Peking Union Medical College, 12 Jiangwangmiao Street, Nanjing, 210042, China; e Department of Neurology, Nanjing Drum Tower Hospital, Affiliated Hospital of Medical School, MOE Key Laboratory of Model Animal for Disease Study, Nanjing University, Nanjing, 210061, China; f Department of Anesthesiology, Hospital of Dermatology, Chinese Academy of Medical Sciences and Peking Union Medical College, 12 Jiangwangmiao Street, Nanjing, 210042, China

**Keywords:** PDK1, Cognitive dysfunction, Myelination, Synaptogenesis, Clemastine

## Abstract

The early postnatal period is a critical window for brain maturation, during which oligodendrogenesis, myelination, and synaptogenesis are dynamically orchestrated to support cognitive development. Clinical studies have associated myelination deficits with intellectual disability (ID), but the molecular mechanisms linking myelin deficits to cognitive dysfunction remain poorly understood. Here, we generated an inducible conditional knockout (icKO) mouse model to selectively ablate 3-phosphoinositide-dependent protein kinase-1 (PDK1) in oligodendrocyte precursor cells (OPCs) during early postnatal development. *Pdk1* icKO mice exhibited severe deficits in hippocampal oligodendrocyte (OL) maturation, myelination, and excitatory synaptogenesis, accompanied by impaired neuronal activation and profound memory impairments. Mechanistically, PDK1 loss led to suppression of the Akt-mTOR signaling pathway, a critical regulator of OL differentiation and myelination. Strikingly, treatment with clemastine, an FDA-approved pro-myelinating agent, effectively restored oligodendrogenesis, myelination, synaptic integrity, neuronal activity, and cognitive performance in *Pdk1* icKO mice, in part by reactivating Akt-mTOR signaling. Together, these findings identify PDK1 as a pivotal regulator of postnatal myelination and cognitive maturation, establish a mechanistic link between oligodendroglial dysfunction and ID, and highlight clemastine as a promising therapeutic candidate for cognitive disorders associated with myelination deficits.

## Graphical abstract

Clemastine-based treatment ameliorates cognitive impairment and hypomyelination in the oligodendroglial PDK1 deficiency mouse model.

**Figure:**
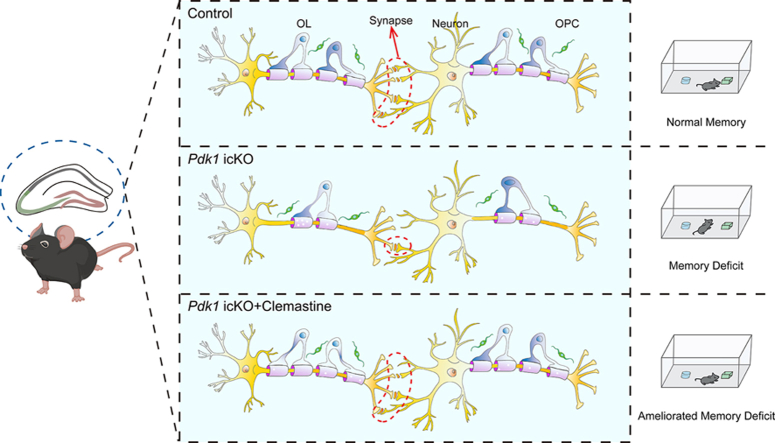

## Introduction

ID is a prevalent neurodevelopmental disorder characterized by persistent deficits in learning, memory, and adaptive functioning, imposing a substantial burden on individuals, families, and healthcare systems [1]. Affecting 2–3% of the global population, ID remains a major unmet medical challenge with limited therapeutic options, highlighting an urgent need for mechanistic insights and targeted interventions [2]. While genetic studies have identified numerous causative mutations, the neurobiological pathways linking genetic risk to cognitive dysfunction remain poorly understood, particularly those involving white matter (WM) development, a critical yet underexplored contributor to ID pathology [[3], [4], [5]].

Among brain regions, the hippocampus is especially vulnerable due to its prolonged postnatal maturation, during which myelination, synapse formation, and circuit refinement are dynamically regulated [[6], [7], [8], [9]]. Disruptions in these processes can lead to long-lasting cognitive impairments, highlighting the hippocampus as a sensitive and functionally pivotal structure in neurodevelopmental disorders [10,11]. However, the molecular regulators coordinating postnatal hippocampal myelination and synaptic organization, and their potential as druggable targets, remain largely undefined.

OLs, derived from OPCs, play pivotal roles in axonal myelination, ensuring efficient neurotransmission and network synchronization [[12], [13], [14]]. Emerging evidence suggests that myelination actively modulates synaptogenesis and functional circuit development [4,5], while excitatory synapses establish the synaptic plasticity necessary for learning and memory [15]. However, the molecular pathways integrating these processes, and their clinical relevance to ID-associated cognitive maturation, are unclear.

PDK1, a key serine/threonine kinase downstream of PI3K signaling, regulates diverse cellular processes, including proliferation, differentiation, metabolism, and survival [16]. Critically, human genetic studies have identified PDK1 mutations in patients with ID and severe brain malformations, strongly implicating PDK1 dysfunction in cognitive impairment [17,18]. Correspondingly, preclinical studies of murine models further support PDK1's role in neurodevelopmental events such as neuronal migration [19], neural progenitor dynamics [20], and interneuron survival [21]. Yet, the role of PDK1 in postnatal hippocampal myelination and synaptic maturation, as well as its therapeutic potential for cognitive function, remains unknown.

Our previous work demonstrated that *Olig1-Cre*-mediated deletion of PDK1 results in profound OL loss and severe myelination defects in the cortex starting from postnatal day 7 (P7), highlighting the essential role of PDK1 in OL development [22]. Moreover, these cKO mice exhibited early postnatal death, with the majority dying before P21, which precluded investigation into the role of oligodendrocytic PDK1 in cognitive functions. To overcome this limitation, we employed an inducible *NG2-CreERT2* system to delete *Pdk1* in OL lineage cells, including OPCs and OLs, during a narrow postnatal window (P10–P12). This approach circumvents potential developmental confounds associated with constitutive knockout and allows us to uncover a critical requirement for PDK1 in hippocampal myelination and cognitive functions.

In the present study, we examined the impact of postnatal PDK1 loss on hippocampal myelination, synaptic integrity, neuronal activity, and cognitive function. We found that *Pdk1* ablation impaired hippocampal oligodendrogenesis, myelination, disrupted excitatory synapses, and led to learning and memory deficits, mirroring core features of ID. Strikingly, pharmacological intervention with clemastine, an FDA-approved antihistamine with pro-myelinating properties, rescued both cellular and cognitive deficits, offering direct preclinical evidence for repurposing myelination-enhancing drugs in ID treatment.

Collectively, our findings establish PDK1 as a critical molecular hub linking hippocampal myelination and synaptic organization to cognition. Moreover, the successful rescue with clemastine provides proof-of-concept support for targeting OL dysfunction as a translatable strategy in ID and related neurodevelopmental disorders. Given that clemastine is already clinically approved, our work may facilitate rapid therapeutic translation, offering an opportunity for patients with cognitive impairments associated with impaired myelination.

## Materials and methods

### Animal models and treatments

*Pdk1*^*fl/fl*^ mice [23] *NG2-CreERT2* mice [24] were obtained for conditional recombination studies as previously described. To generate conditional knockout mice, *Pdk1*^*fl/fl*^ mice were crossed with *NG2-CreERT2* mice to produce *Pdk1*^*fl/+*^; *NG2-CreERT2* offspring, which were subsequently backcrossed with *Pdk1*^*fl/fl*^ mice to obtain *Pdk1*^*fl/fl*^; *NG2-CreERT2* (*Pdk1* icKO) mice. Littermate *Pdk1*^*fl/fl*^ mice were used as controls.

For inducible recombination, tamoxifen (50 mg/kg) was administered once daily from P10 to P12. For pharmacological rescue, clemastine fumarate (10 mg/kg), a first-generation antihistamine with established remyelination-promoting effects, was administered once daily from P21 to P58. All mice were maintained on a C57BL/6 background and included both sexes. Animals were housed under standard laboratory conditions (24 ± 1 °C; 12-h light/dark cycle) with free access to food and water. All experimental procedures were reviewed and approved by the Institutional Animal Care and Use Committee (IACUC) of the Model Animal Research Center (MARC), Nanjing University (Animal Protocol License Number: 220203203), and were conducted in compliance with the guidelines established by the Jiangsu Association for Laboratory Animal Science (JSALAS).

### Y-maze test

Spatial working memory was assessed using the Y-maze spontaneous alternation paradigm, as previously described [25]. Mice were habituated to the behavioral testing room for 30 min prior to testing. Each mouse was placed at the distal end of one randomly selected arm and allowed to freely explore the maze for 8 min. Arm entries and alternations were recorded with an overhead video-tracking system. The percentage of spontaneous alternation was calculated using the formula: %Alternation = (Number of Alternations/[Total number of arm entries - 2]) x 100.

### Novel object recognition (NOR) test

Recognition memory was evaluated using the novel object recognition test, as previously described [26]. Mice were habituated to the behavioral testing room for 30 min prior to testing. During the familiarization phase, each mouse was placed in the arena and allowed to explore two identical objects for 5 min before being returned to the home cage. After a 3-h retention interval, one of the familiar objects was replaced with a novel object of similar size but different shape and texture. Mice were then returned to the arena and allowed to explore freely for 5 min. The discrimination index (DI) was calculated using the following formula: DI = T_n_/(T_n_ + T_f_), where T_n_ represents the time spent exploring the novel object and T_f_ denotes the time spent exploring the familiar object during the testing phase.

### BrdU labeling assay

For proliferation analysis, mice received a single intraperitoneal injection of BrdU (100 mg/kg) at P13 and were sacrificed 2 h later. Hippocampal sections were stained for BrdU and Olig2, and BrdU^+^/Olig2^+^ cells were quantified to evaluate OPC proliferation. For differentiation analysis, mice were injected with BrdU daily from P13 to P15 and sacrificed at P28. Sections were stained for BrdU and CC1, and the proportion of BrdU^+^/CC1^+^ cells was quantified to assess differentiation.

### Western blotting

Mouse hippocampal tissues were rapidly dissected and snap-frozen in liquid nitrogen. Samples were homogenized in ice-cold RIPA lysis buffer (50 mM Tris-HCl, pH 7.4; 150 mM NaCl; 1% NP-40; 0.5% sodium deoxycholate; 0.1% SDS) supplemented with protease and phosphatase inhibitor cocktails. Lysates were centrifuged at 4 °C for 15 min, and the supernatant was collected. Protein concentration was determined using the bicinchoninic acid (BCA) assay. Equal amounts of protein (40 μg per lane) were separated by SDS-PAGE and transferred onto nitrocellulose membranes. Membranes were blocked with 5% non-fat dry milk for 60 min at room temperature and incubated with primary antibodies at 4 °C overnight, followed by incubation with IRDye-conjugated secondary antibodies at room temperature for 60 min. The following primary antibodies were used: PDK1 (Abcam, Cat# ab52893, 1:500), Akt (Cell Signaling Technology, Cat# 4691, 1:1000), p-Akt^Thr308^ (Cell Signaling Technology, Cat# 13038, 1:1000), p-S6^Ser235/236^ (Cell Signaling Technology, Cat# 2211, 1:1000), S6 (Abclonal, Cat# A6058, 1:1000), and β-actin (GenTex, Cat# CTX124212, 1:5000). Protein bands were visualized and quantified using the Li-COR Odyssey imaging system.

### Quantitative real-time PCR (qRT-PCR)

Total RNA was extracted from hippocampal tissues using TRIzol reagent (Takara Bio, Cat# 9108). Reverse transcription was performed with the HiScript II Q RT SuperMix for qPCR (+gDNA wiper) (Vazyme, Cat# R223-01) according to the manufacturer's instructions. qRT-PCR was carried out using ChamQ SYBR qPCR Master Mix (Vazyme, Cat# Q311-02) on a Roche LightCycler system. Thermal cycling conditions consisted of an initial denaturation at 95 °C for 3 min, followed by 40 cycles of denaturation at 95 °C for 10 s and annealing/extension at 60 °C for 20 s. Relative gene expression was quantified using the comparative Ct method. *Gapdh* was used as an internal control. Primer sequences are provided in the Supplementary Table.

### Transmission electron microscopy (TEM)

Mice were deeply anesthetized with 2% isoflurane and transcardially perfused with PBS, followed by fixation with 2.5% glutaraldehyde in 0.1 M phosphate buffer (pH 7.4). Hippocampal tissues were carefully dissected at P21 and P28 and post-fixed in the same fixative at 4 °C overnight. Samples were subsequently processed by Lilai Biological Technology Co., Ltd. (Chengdu, China) using standard TEM procedures, including post-fixation with 1% osmium tetroxide, dehydration through a graded ethanol series, and embedding in epoxy resin. Ultrathin sections were prepared and stained with uranyl acetate and lead citrate. Sections were examined under a transmission electron microscope, and representative images were acquired for ultrastructural analysis. Axon diameter, myelin thickness, and *g*-ratio (defined as the ratio of the inner axonal diameter to the total fiber diameter) were quantified using ImageJ software.

### Electrophysiology

Miniature excitatory postsynaptic current (mEPSC) recordings were performed as previously described [27]. Briefly, hippocampi were sectioned into 350 μm slices using a vibratome (VT1000 S, Leica) in ice-cold high-sucrose cutting solution (in mM: KCl 2.5, NaH_2_PO_4_ 1.25, NaHCO_3_ 25, CaCl_2_ 0.5, MgSO_4_ 7, sucrose 210, D-glucose 10, and Na-ascorbate 1.3; all from Sigma-Aldrich, USA), which was oxygen-saturated with 95% O_2_/5% CO_2_). Slices were incubated in oxygen-saturated artificial cerebrospinal fluid (ACSF; in mM: NaCl 119, KCl 2.5, CaCl_2_ 2.5, MgSO_4_ 1.3, NaHCO_3_ 26.2, NaH_2_PO_4_ 1, and D-glucose 11) at 32 °C for 20 min and then at room temperature for 60 min for recovery before recording.

Hippocampal slices were transferred to a recording chamber and continuously perfused with ACSF supplemented with the GABAA receptor antagonists picrotoxin (PTX, 100 μM; Tocris, UK) and bicuculline (Bic, 10 μM; Absin, China), as well as the NaV channel blocker tetrodotoxin (TTX, 1 μM, Absin, China). Recording electrodes (3–6 MΩ) were filled with internal solution (in mM: CsMeSO_3_ 135, NaCl 8, HEPES 10, EGTA 0.3, Mg-ATP 4, Na_3_-GTP 0.3, QX-314 5, and spermine 0.1; 295 mOsm/L; pH7.3 adjusted with CsOH). Whole-cell patch-clamp recordings were obtained from pyramidal neurons in the CA1 stratum pyramidale. mEPSCs were recorded at a holding potential of −70 mV in gap-free mode for 10 min.

Electrophysiological signals were recorded with a Multiclamp 700B amplifier and a Digidata 1550 data acquisition system (Axon Instruments, USA), filtered at 2 kHz, and digitized at 50 kHz.

### Immunofluorescence staining (IHC)

Adult mouse brains were dissected after perfusion with ice-cold phosphate-buffered saline (PBS) and fixed in 4% paraformaldehyde (PFA) at 4 °C overnight. Samples were then dehydrated in 30% sucrose at 4 °C and sectioned at 10 μm thickness using a freezing microtome. For antigen retrieval, sections were boiled in citrate buffer (pH 6.0) for 25 min. After blocking with 5% bovine serum albumin (BSA), sections were incubated with primary antibodies at 4 °C overnight, followed by incubation with secondary antibodies at room temperature for 60 min. The following primary antibodies were used: MBP (Millipore, Cat# MAB386, 1:500), Olig2 (Millipore, Cat# MABN50, 1:500), NeuN (Millipore, Cat# ABN78, 1:500), GFAP (Santa Cruz, Cat# sc-65343, 1:500), VGlut1 (Synaptic Systems, Cat# 135303, 1:500), VGAT (Synaptic Systems, Cat# 131003, 1:300), Homer1 (Synaptic Systems Cat# 160003, 1:500), PSD95 (PSD95, Abcam, Cat# ab18258, 1:500), MAP2 (Millipore, Cat# MAB3418, 1:500), c-Fos (Oasis, Cat# OB-PGP080, 1:500), PDGFRα (Cell Signaling Technology, Cat# 3174, 1:500), CC1 (Calbiochem, Cat# op-80, 1:300) and ASPA (Millipore, Cat# ABN1698, 1:500). Nuclei were counterstained with DAPI (Sigma-Aldrich, Cat# D9542, 1 mg/ml). Fluorescence images were acquired using an Olympus BX53 or SS-MCS microscope. Fluorescence images were acquired using an Olympus BX53 or SS-MCS microscope. For image quantification, the entire hippocampal region was delineated as the region of interest (ROI) based on DAPI staining using ImageJ software. For cell density analysis (e.g., Olig2, PDGFRα), a total census of immunoreactive cells within the entire hippocampal ROI was performed.

### TrueGold myelin staining

Gross myelin was examined using TrueGold staining according to the manufacturer's instructions. Brain sections (10 μm thickness) were prepared as described above and incubated in TrueGold solution for 30 min [28]. Sections were rinsed 3 times with distilled water to remove excess dye. Brightfield images were acquired using an Olympus BX53 microscope. For each region of interest, at least 3 fields were imaged at 20× magnification. Quantification of myelination (for both MBP and TrueGold) was performed by calculating the area fraction. Briefly, images were converted to 8-bit grayscale, and a consistent, pre-determined threshold was applied to identify myelin-positive signals. The percentage of the area covered by positive signals within the total hippocampal ROI was then measured. To minimize inter-batch variance, the absolute area fraction of each section was normalized to the mean of the control group, and data were expressed as “Relative area of MBP” or “Relative area of myelin.”

### Statistical analysis

Data were presented as mean ± SEM. Unpaired t-tests were applied for comparisons between two groups, while one-way ANOVA and two-way ANOVA were used for multiple group comparisons depending on experimental design complexity. For each analysis, at least 4 mice per group and 3 equidistant sections (approximately Bregma −1.70 to −2.30 mm) per mouse were included. The values from the three sections were averaged for each animal to ensure that the individual mouse (n ≥ 4) served as the experimental unit for statistical comparisons. All statistical analyses were performed using GraphPad Prism 8 software. All histological quantifications were performed by an investigator blinded to the experimental genotypes and treatment groups.

For each analysis, at least 4 mice per group and 3 sections per mouse were included. All statistical analyses were performed using GraphPad Prism 8 software.

## Results

### Postnatal PDK1 deficiency in OPCs leads to hippocampal hypomyelination and cognitive deficits

To investigate the role of PDK1 in postnatal myelination and cognitive development, *Pdk1*^*fl/fl*^ mice [22] were crossed with *NG2-CreERT2* mice [24] to generate *Pdk1*^*fl/fl*^; *NG2-CreERT2* (*Pdk1* icKO) mice. Tamoxifen was administered from P10–P12, a critical window for hippocampal oligodendrogenesis and myelination. Analyses were conducted at P60 to assess long-term effects (Fig. 1A). qRT-PCR analysis revealed a significant reduction of *Pdk1* mRNA in the hippocampus of *Pdk1* icKO mice compared with controls (Fig. 1B). Western blotting confirmed a pronounced decrease in PDK1 protein levels (Fig. 1C and D), demonstrating efficient postnatal ablation of PDK1 in hippocampal OPCs. IHC for MBP showed a marked reduction in myelin area in the hippocampal CA1 and dentate gyrus (DG) of *Pdk1* icKO mice (Fig. 1E and F). TrueGold staining [28] further corroborated these findings, revealing a pronounced decrease in overall hippocampal myelin content in *Pdk1* icKO mice (Fig. 1G and H).

**Fig. 1:**
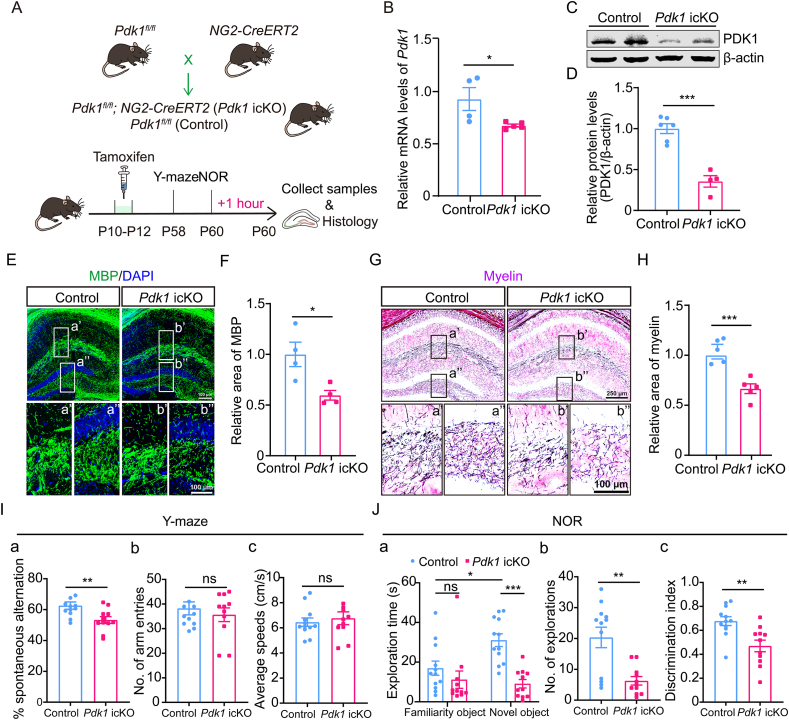
**Hippocampal hypomyelination and cognitive deficits in *Pdk1* icKO mice** (A) Mouse breeding strategy and experimental schedule. *NG2-CreERT2* mice were crossed with *Pdk1*^*fl/fl*^ mice to obtain *Pdk1* icKO mice or control littermates. Tamoxifen was administered from P10 to P12 to induce Cre-mediated recombination. Behavioral tests (Y-maze and NOR) were conducted at P58–P60, followed by tissue collection. (B) qRT-PCR analysis of *Pdk1* mRNA expression in control and *Pdk1* icKO hippocampal tissues at P60. *Pdk1* mRNA levels were significantly reduced in the *Pdk1* icKO hippocampus. The results were shown as mean ± SEM (n = 4–5 per genotype; ∗, *p* < 0.05). (C, D) Western blotting analysis for PDK1 in the hippocampal lysates of control and *Pdk1* icKO mice. The relative protein levels of PDK1 were largely decreased in the *Pdk1* icKO mice. β-actin served as a loading control. Data were shown as mean ± SEM (n = 4–6 per genotype; ∗∗∗, *p* < 0.001). (E) Representative IHC images of MBP (green) in the brain sections of control and *Pdk1* icKO mice at P60. Higher-magnification images of the corresponding boxed hippocampal areas are displayed in panels a', a'', b', and b''. Cellular nuclei are identified by DAPI (blue) counterstaining. Scale bar: 100 μm. (F) Statistical analysis of MBP immunofluorescence intensity within the hippocampus. The intensity was markedly lower in *Pdk1* icKO mice than in controls, indicating impaired myelination. Values are expressed as mean ± SEM (n = 4 per genotype; ∗, *p* < 0.05). (G, H) Representative images of TrueGold-stained brain sections from control and *Pdk1* icKO mice at P60. The hippocampal sub-regions within the boxed areas are shown at higher magnification (a', a'', b', b''). Quantification revealed a significant reduction in TrueGold staining intensity in the hippocampus of *Pdk1* icKO mice compared to controls. Data are shown as mean ± SEM (n = 5 per genotype; ∗∗∗, *p* < 0.001). Scale bars: 250 μm (low magnification), 100 μm (high magnification). (I) Y-maze test. The *Pdk1* icKO group showed a significant reduction in spontaneous alternation compared to control mice. Data were shown as mean ± SEM (n = 11–12 per genotype; ∗∗, *p* < 0.01). (J) NOR test. *Pdk1* icKO mice exhibited reduced exploration time and fewer exploration events toward the novel object. Consequently, the discrimination index was significantly lower in *Pdk1* icKO mice than in controls. Data were shown as mean ± SEM (n = 11–12 per genotype; ∗∗, *p* < 0.01).

To determine whether PDK1 deficiency affects myelin ultrastructure, we performed TEM analysis using brain tissues prepared from mice at P21 and P28, representing two different stages of postnatal myelination. Ultrastructural examination revealed normally compacted myelin sheaths without overt dysmorphic features in *Pdk1* icKO mice (Fig. S1A and C). However, *Pdk1* icKO mice exhibited significantly thinner myelin sheaths compared with controls, as evidenced by increased *g*-ratios (Fig. S1B and D). These findings indicate that postnatal PDK1 ablation results in hippocampal hypomyelination.

Behavioral analyses were then conducted to assess whether these myelination defects impacted cognitive function. In the Y-maze test, *Pdk1* icKO mice exhibited significantly reduced spontaneous alternation rates relative to controls, indicating impaired spatial working memory (Fig. 1Ia). No differences were observed in total arm entries or average locomotor speed (Fig. 1Ib and c), suggesting preserved general locomotor activity. In the NOR test, *Pdk1* icKO mice showed decreased exploration of the novel object (Fig. 1Ja and b) and a significantly reduced discrimination index (Fig. 1Jc), indicative of impaired recognition memory.

To further explore the regional specificity of PDK1-mediated myelination, we extended our IHC analysis to other central nervous system (CNS) regions. While significant demyelination was observed in the prefrontal cortex (PFC), the cerebellar myelin integrity remained comparable to controls (Fig. S1E and F). These structural findings align with the cognitive impairments observed in Y-maze and NOR tests, which are primarily mediated by hippocampal and cortical circuits [29,30], while the spared cerebellar myelin is consistent with the intact motor performance of *Pdk1* icKO mice.

Collectively, these results demonstrate that postnatal PDK1 deletion in OPCs suppresses myelination and impairs both spatial and recognition memory, highlighting the critical role of PDK1 in postnatal oligodendrogenesis and cognitive function.

### Loss of PDK1 in OPCs impairs hippocampal oligodendrogenesis

To investigate the mechanisms underlying hippocampal hypomyelination in *Pdk1* icKO mice, we systematically analyzed oligodendrogenesis in the hippocampus. IHC staining for Olig2, a pan-OL lineage marker, revealed a significant reduction in Olig2-positive (+) cells in *Pdk1* icKO mice compared to controls (Fig. 2A and B), indicating a decrease in overall OL lineage cell populations. Next, OPCs were examined by IHC for PDGFRα. The immunoreactivity and cell density of PDGFRα^+^ cells were comparable between *Pdk1* icKO and control mice (Fig. 2C and D), suggesting that early-stage OPC proliferation or maintenance was largely unaffected by PDK1 deletion. We then assessed mature OLs using anti-adenomatous polyposis coli (APC) clone (CC1) and anti-aspartoacylase (ASPA) antibody, the well-established marker of myelinating OLs [31,32]. IHC analysis revealed a marked decrease in CC1^+^ and ASPA^+^ immunoreactivity in the hippocampus of *Pdk1* icKO mice (Fig. 2E and S2A), and quantitative cell counting confirmed a significant reduction in CC1^+^ and ASPA^+^ cell density (Fig. 2F and S2B). These results indicate that postnatal PDK1 ablation selectively impairs OL maturation rather than OPC maintenance.

**Fig. 2:**
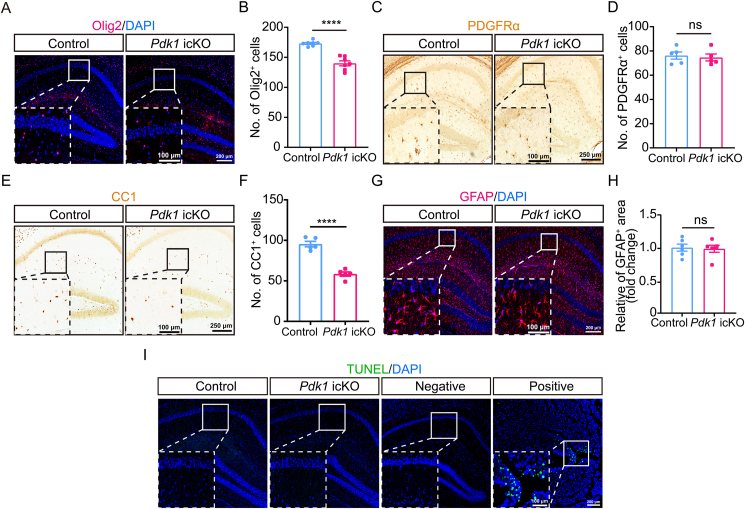
**Reduced hippocampal oligodendrogenesis in *Pdk1* icKO mice** (A, B) Representative IHC images for Olig2 (red) in brain sections from control and *Pdk1* icKO mice. Nuclei were counterstained with DAPI (blue). A significant decrease in the number of Olig2^+^ cells was observed in *Pdk1* icKO mice compared with controls. Data are presented as mean ± SEM (n = 7 per genotype; ∗∗∗∗, *p* < 0.0001). Scale bars: 200 μm (overview), 100 μm (magnified views). (C, D) Representative IHC images of PDGFRα expression in brain sections from control and *Pdk1* icKO mice. No significant difference in PDGFRα+ cell density was detected between genotypes. Data are presented as mean ± SEM (n = 5 per genotype; ns, not significant). Scale bars: 250 μm (overview), 100 μm (magnified views). (E) IHC staining for CC1 in brain sections of control and *Pdk1* icKO mice. Scale bars: 200 μm (overview), 100 μm (magnified views). (F) Quantification of CC1^+^ cells revealed a significant reduction in *Pdk1* icKO mice compared to controls. Data are presented as mean ± SEM (n = 6–7 per genotype; ∗∗∗, *p* < 0.001). (G, H) Representative IHC images of GFAP (red) staining in brain sections from control and *Pdk1* icKO mice. Nuclei were counterstained with DAPI (blue). No significant difference in GFAP expression was observed between genotypes. The results were shown as mean ± SEM (n = 6 per genotype; ns, not significant). Scale bars: 200 μm (overview), 100 μm (magnified views). (I) TUNEL (green) assay performed on brain sections from control and *Pdk1* icKO mice. Nuclei were counterstained with DAPI (blue). No significant difference in the number of TUNEL^+^ cells was detected in the hippocampus between genotypes. Scale bars: 200 μm (overview), 100 μm (magnified views).

Given that oligodendrogenesis and astrogenesis occur concurrently during early postnatal brain development [33], we examined whether PDK1-dependent OL deficits affect astrocyte development. IHC for GFAP showed no significant difference in GFAP^+^ area between *Pdk1* icKO and control mice (Fig. 2G and H), suggesting that astrogenesis remained intact. Considering the established role of PDK1 in cell survival [34], we further investigated whether apoptotic cell death contributes to the observed reduction in mature OLs. TUNEL staining was performed on hippocampal sections, with *Ppp2cα* knockout mouse brain sections serving as a positive control [35]. While abundant TUNEL-labeled cells were detected in the positive control, no TUNEL-labeled cells were observed in the hippocampus of *Pdk1* icKO mice (Fig. 2I), indicating that PDK1 deficiency in OPCs does not induce apoptosis.

Together, these data suggest that postnatal loss of PDK1 in OPCs specifically disrupts OL maturation in the hippocampus without affecting OPC proliferation, astrocyte development, or cell survival.

### Postnatal PDK1 deletion impairs OPC differentiation without affecting proliferation

While our steady-state analysis of PDGFRα^+^ cells suggested a maintained OPC pool, it remained unclear whether the reduction in mature OLs was due to subtle defects in OPC proliferation dynamics or a primary failure in their differentiation into mature myelinating cells. To rigorously distinguish between these possibilities, we performed BrdU birth-dating experiments (Fig. S2C–F). We first assessed active OPC proliferation by administering a single pulse of BrdU at P13. 24 h after the final tamoxifen induction, we prepared brain sections from these mice at 2 h post-injection. Immunostaining for Olig2 and BrdU revealed that the proportion of proliferating oligodendroglial lineage cells (BrdU^+^/Olig2^+^) in the hippocampus was comparable between control and *Pdk1* icKO mice (Fig. S2C and D). These results indicate that postnatal PDK1 deletion does not impair OPC proliferative activity in the CNS. Next, we evaluated the differentiation potential of newly generated OPCs using a pulse-chase paradigm. Mice were injected with BrdU from P13 to P15 and sacrificed at P28, a stage at which a substantial fraction of early-born OPCs is expected to have matured into CC1^+^ myelinating OLs. Quantitative analysis revealed a significant reduction in the proportion of BrdU^+^/CC1^+^ cells relative to total CC1^+^ OLs in *Pdk1* icKO mice compared with controls (Fig. S2E and F), indicating impaired differentiation of newly generated OPCs.

To determine whether PDK1 deletion disrupts transcriptional programs governing OL differentiation, we examined the expression of key lineage-specific transcription factors. *Sox10* serves as a master regulator of OL lineage specification and progression, whereas *Myrf* is a critical driver of terminal differentiation and myelin gene activation [32,36,37]. Our qRT-PCR analysis demonstrated a significant reduction in *Sox10* and *Myrf* mRNA levels in the hippocampus of *Pdk1* icKO mice compared with control littermates (Fig. S2G). Their coordinated downregulation provides molecular evidence that PDK1 deficiency impairs the transcriptional network required for OL maturation rather than affecting progenitor proliferation.

In contrast to the constitutive *Pdk1* cKO mouse model (*Pdk1*^*fl/fl*^;*Olig1-Cre*) [22], *Pdk1* icKO mice remained viable and did not exhibit spontaneous seizures or early lethality. These data collectively demonstrate that the postnatal loss of PDK1 specifically arrests the transition of OPCs into mature, myelinating OLs, leading to the chronic hypomyelination and cognitive deficits observed in adult mice, without triggering the catastrophic global neurodevelopmental failure associated with embryonic PDK1 loss.

### Preservation of axonal integrity despite hypomyelination in *Pdk1* icKO mice

To determine whether impaired OL maturation and subsequent hypomyelination led to secondary axonal degeneration, we performed IHC staining for neurofilament 200 (NF200), a marker of axons, and DAPI in the hippocampus of control and *Pdk1* icKO mice. Detailed morphological analysis revealed that the NF200-labeled axons in *Pdk1* icKO mice did not exhibit typical pathological features of degeneration, such as axonal fragmentation, focal swelling, or beading (Fig. S3). These results demonstrate that the axons remain structurally intact but hypomyelinated. Thus, the observed cognitive deficits are likely functional consequences of impaired myelination rather than a result of irreversible axonal loss.

### Deletion of PDK1 in OPCs impairs hippocampal excitatory synaptogenesis and neuronal activity

Given that myelination and synaptogenesis are temporally coupled during early postnatal development [38] and that disrupted myelination can lead to synaptic loss [39], we next examined whether PDK1 deficiency-mediated hypomyelination affects synapse formation. Excitatory synapses were assessed by co-immunostaining for MAP2, a neuronal marker, and VGlut1, a presynaptic excitatory terminal marker. *Pdk1* icKO mice exhibited a significant reduction in VGlut1^+^ puncta adjacent to MAP2^+^ neurons in the hippocampus compared to littermate controls (Fig. 3A and B), indicating that excitatory synaptogenesis is partially compromised. Consistently, Homer1^+^ postsynaptic puncta were significantly reduced in *Pdk1* icKO mice (Fig. 3C and D), and Western blot analysis revealed decreased PSD95 protein levels (Fig. S4A and B), further supporting impaired excitatory synapse formation. In contrast, inhibitory synapses, evaluated by co-immunostaining for MAP2 and VGAT (a presynaptic GABAergic marker), showed no significant differences between *Pdk1* icKO and control mice (Fig. S4C and D), suggesting that PDK1 deficiency selectively affects excitatory, but not inhibitory, synapses in the hippocampus.

**Fig. 3:**
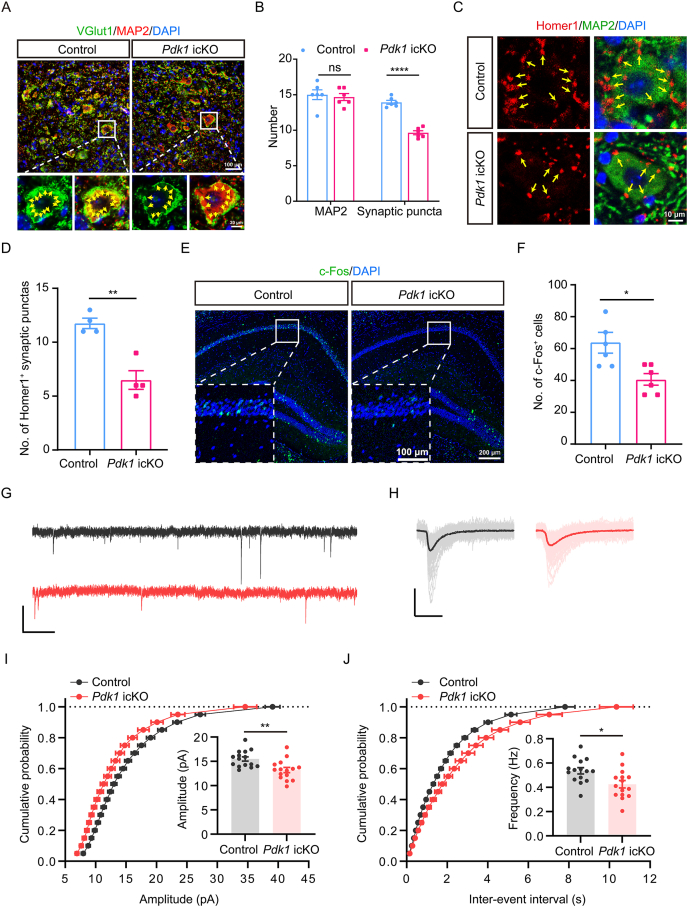
**PDK1 deletion impairs excitatory synaptogenesis and synaptic transmission in the hippocampus** (A) Representative IHC images of hippocampal sections co-stained for MAP2 (red) and VGlut1 (green) in *Pdk1* icKO and control mice. Nuclei are counterstained with DAPI (blue). Arrows indicate VGlut1^+^ presynaptic puncta. Scale bars: 100 μm (overview), 20 μm (magnified views). (B) Quantification of synaptic puncta revealed a significant reduction in the density of VGlut1^+^ puncta along MAP2^+^ dendrites in the hippocampus of *Pdk1* icKO mice. Data are presented as mean ± SEM (n = 6 per genotype; ∗∗∗∗, *p* < 0.0001). (C) Representative images of Homer1 (red), MAP2 (green), and DAPI (blue) staining in the hippocampus. Arrows indicate Homer1^+^ postsynaptic puncta. Scale bar: 10 μm. (D) Quantification of Homer1^+^ synaptic puncta showing a significant reduction in *Pdk1* icKO mice. Data are presented as mean ± SEM (n = 4 per genotype; ∗∗, *p* < 0.01). (E) Representative images of c-Fos (green) immunostaining in brain sections from control and *Pdk1* icKO mice. Nuclei are counterstained with DAPI (blue). Scale bars: 200 μm (overview), 100 μm (magnified views). (F) Quantification of c-Fos^+^ cells showed a significant decrease in the hippocampus of *Pdk1* icKO mice compared to controls. Data are presented as mean ± SEM (n = 6 per genotype; ∗, *p* < 0.05). (G) Represented mEPSC traces in control (black) and *Pdk1* icKO (red) mice. Scale bar: 20 mV, 2 s. (H) Averaged mEPSC traces from control (black) and *Pdk1* icKO (red) neurons. Aligned mEPSC peaks and the average trace are shown in dark color from a typical neuron. Scale bar: 20 mV, 20 ms. (I) Cumulative probability plot and quantification of mEPSC amplitudes. *Pdk1* icKO mice exhibited a significant reduction in mEPSC amplitude compared to controls. Data are presented as mean ± SEM (n = 3 per genotype; ∗∗, *p* < 0.01). (J) Cumulative probability plot and quantification of mEPSC inter-event intervals. mEPSC frequency was significantly decreased in *Pdk1* icKO mice. Data are presented as mean ± SEM (n = 3 per genotype; ∗, *p* < 0.05).

To further assess neuronal activity *in vivo*, hippocampal tissues were collected from mice 60 min following behavioral testing. The number of NeuN^+^ neurons did not differ between genotypes (Fig. S4E and F), indicating preserved neuronal density. However, immunostaining for c-Fos, a marker of neuronal activation, revealed markedly reduced immunoreactivity in *Pdk1* icKO mice (Fig. 3E). Quantitative analysis confirmed a significant decrease in c-Fos^+^ cell density in the hippocampus of *Pdk1* icKO mice relative to controls (Fig. 3F), indicating diminished neuronal activation following PDK1 deletion.

To determine whether these structural synaptic alterations translate into functional deficits, we recorded mEPSCs in hippocampal neurons using whole-cell patch-clamp. Representative traces are shown in Fig. 3G and H. Quantitative analysis revealed a significant reduction in the average amplitude of mEPSCs in *Pdk1* icKO mice compared to controls (Fig. 3I), indicating reduced postsynaptic strength. Moreover, mEPSC frequency was also significantly reduced in *Pdk1* icKO mice (Fig. 3J), suggesting a reduced number of functional excitatory synapses. These findings demonstrate that PDK1 deficiency impairs transmission of excitatory synapses.

Together, these results demonstrate that postnatal PDK1 ablation in OPCs leads to impaired excitatory synaptogenesis, reduced synaptic transmission, and decreased neuronal activation in the hippocampus. Considering the well-established role of hippocampal excitatory transmission in spatial working memory and recognition memory [40], these synaptic and functional deficits likely contribute to the cognitive impairments observed in *Pdk1* icKO mice.

### Clemastine rescues hippocampal hypomyelination and synaptic deficits in *Pdk1* icKO mice

**Fig. 4:**
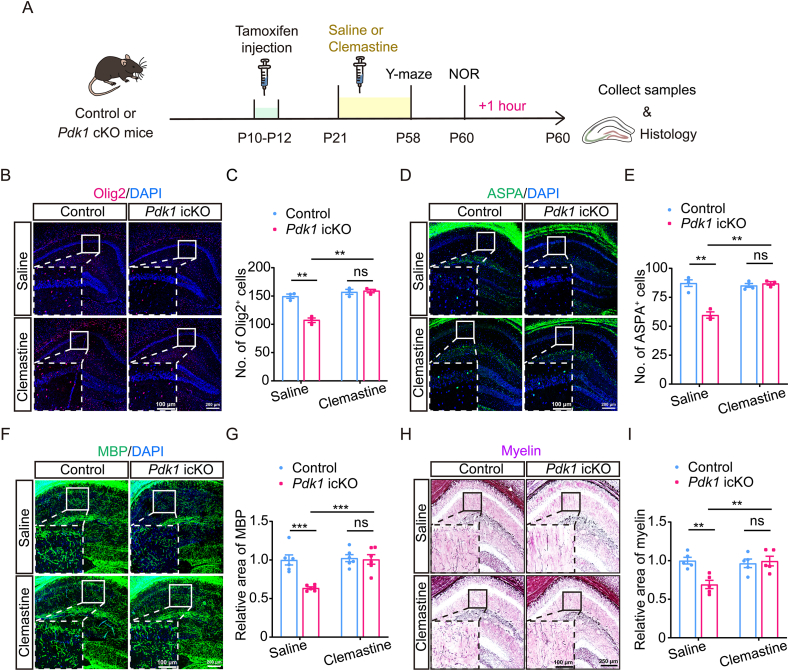
**Clemastine treatment rescues impaired hippocampal oligodendrogenesis and myelination in *Pdk1* icKO mice** (A) Experimental timeline. Tamoxifen was administered from P10 to P12 to induce knockout, followed by clemastine treatment from P21 to P58. Behavioral tests (Y-maze and NOR) were performed before brain tissue collection at P60. (B) Representative IHC images of Olig2^+^ (red) cells in brain sections from control and *Pdk1* icKO mice treated with saline or clemastine. Nuclei were counterstained with DAPI (blue). Scale bars: 200 μm (overview), 100 μm (magnified views). (C) Quantification of Olig2^+^ cells. Clemastine treatment significantly increased the number of Olig2^+^ cells in *Pdk1* icKO mice compared to saline-treated mutants. Data are presented as mean ± SEM (n = 3 per group; ∗∗, *p* < 0.01). (D) IHC staining for ASPA (green) in brain sections from the indicated experimental groups. Nuclei were counterstained with DAPI (blue). Scale bars: 200 μm (overview), 100 μm (magnified views). (E) Quantification of ASPA^+^ cells. Clemastine-treated *Pdk1* icKO mice showed a significant increase in ASPA^+^ cell density compared to saline-treated mutants. Data are presented as mean ± SEM (n = 3–4 per group; ∗∗, *p* < 0.01). (F) Representative IHC images of MBP (green) expression in brain sections from control and *Pdk1* icKO mice treated with saline or clemastine. Nuclei were counterstained with DAPI (blue). Scale bars: 200 μm (overview), 100 μm (magnified views). (G) Quantification of the MBP^+^ area. Clemastine treatment significantly increased the relative MBP^+^ area in *Pdk1* icKO mice compared to those treated with saline. Data are presented as mean ± SEM (n = 5 per group; ∗∗∗, *p* < 0.001). Scale bars: 250 μm (overview), 100 μm (magnified views). (H, I) Representative images of TrueGold-stained brain sections from control and *Pdk1* icKO mice treated with saline or clemastine at P60. Quantification revealed a significant increase in TrueGold staining intensity in the hippocampus of *Pdk1* icKO mice treated with clemastine. Data are shown as mean ± SEM (n = 5 per genotype; ∗∗, *p* < 0.001). Scale bars: 250 μm (low magnification), 100 μm (high magnification).

Clemastine, an FDA-approved antihistamine, has recently been identified as a pro-myelination agent that promotes OL differentiation and myelin formation [41,42]. To test whether clemastine could rescue myelination deficits induced by PDK1 ablation in OPCs, *Pdk1* icKO mice were treated with clemastine from P21 to P58, and the hippocampus was analyzed at P60 (Fig. 4A). IHC revealed increased Olig2 immunoreactivity in the hippocampus of clemastine-treated *Pdk1* icKO mice (Fig. 4B), and quantitative analysis confirmed a significant restoration of Olig2^+^ cell numbers compared to saline-treated mutants (Fig. 4C). The number of ASPA^+^ mature OLs was also significantly rescued by clemastine treatment (Fig. 4D and E), indicating improved OL maturation. We then evaluated the effects of clemastine on hippocampal myelination. MBP immunostaining demonstrated markedly enhanced signal intensity in clemastine-treated *Pdk1* icKO mice relative to saline-treated controls (Fig. 4F and G). Consistently, TrueGold staining revealed a substantial increase in overall hippocampal myelin density following clemastine administration (Fig. 4H and I). These results indicate that pharmacological enhancement of OL function by clemastine effectively restores both oligodendrogenesis and hippocampal myelination in *Pdk1* icKO mice.

**Fig. 5:**
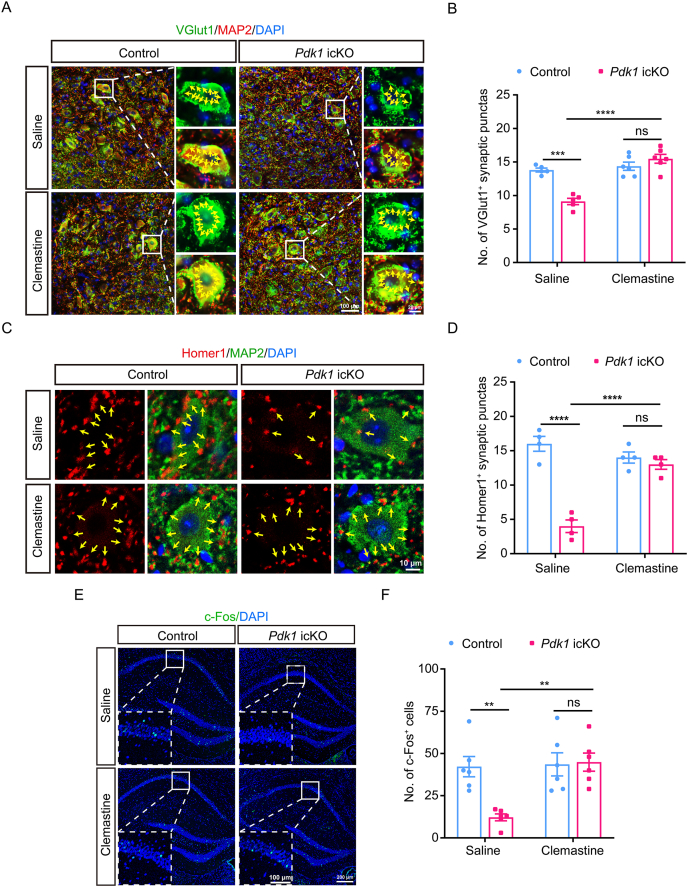
**Clemastine rescues excitatory synaptic deficits and neuronal activity in *Pdk1* icKO mice.** (A) Representative images of hippocampal sections co-immunostained for MAP2 (red) and VGlut1 (green) from control and *Pdk1* icKO mice treated with either saline or clemastine. Nuclei are counterstained with DAPI (blue). Arrows indicate VGlut1^+^ postsynaptic puncta. Scale bars: 100 μm (overview), 20 μm (magnified views). (B) Quantitative analysis of VGlut1^+^ synaptic puncta. Clemastine-treated *Pdk1* icKO mice exhibited a significant increase in the density of VGlut1^+^ puncta along MAP2^+^ neurons compared to saline-treated mutants. Data are presented as mean ± SEM (n = 5–6 per group; ∗∗∗, *p* < 0.001; ∗∗∗∗, *p* < 0.0001). (C) Representative images of hippocampal sections co-immunostained for Homer1 (red), MAP2 (green), and DAPI (blue) from control and *Pdk1* icKO mice treated with saline or clemastine. Arrows indicate Homer1^+^ postsynaptic puncta. Scale bar: 10 μm. (D) Quantification of Homer1^+^ synaptic puncta density. Homer1 expression was significantly reduced in saline-treated *Pdk1* icKO mice and was markedly restored following clemastine treatment. No significant effect of clemastine was observed in control mice. Data are presented as mean ± SEM (n = 4 per group; ∗∗∗∗, *p* < 0.0001). (E) Representative immunostaining images of c-Fos (green) in brain sections from control and *Pdk1* icKO mice treated with saline or clemastine. Nuclei are counterstained with DAPI (blue). Scale bars: 200 μm (overview), 100 μm (magnified views). (F) Quantification of c-Fos^+^ cells revealed a significant increase in the hippocampus of clemastine-treated *Pdk1* icKO mice compared to saline-treated mutants. Data are presented as mean ± SEM (n = 6 per group; ∗∗, *p* < 0.01).

We next examined whether clemastine treatment could rescue synaptic deficits and neuronal activity in the hippocampus of *Pdk1* icKO mice. Co-immunostaining for MAP2 and VGlut1 revealed a significant reduction in excitatory synaptic puncta density in vehicle-treated *Pdk1* icKO mice compared to saline-treated controls, which was effectively restored by clemastine administration (Fig. 5A and B). Notably, clemastine treatment did not significantly alter VGlut1^+^ puncta density in control mice, indicating that its effect was specific to the pathological condition (Fig. 5A and B). To further validate the rescue of postsynaptic structures, we examined Homer1 expression. Consistent with the VGlut1 findings, Homer1^+^ puncta were significantly decreased in saline-treated *Pdk1* icKO mice, whereas clemastine treatment markedly increased Homer1^+^ synaptic puncta to levels comparable to controls (Fig. 5C and D). These data demonstrate that clemastine restores both presynaptic and postsynaptic components of excitatory synapses in the hippocampus.

We next assessed whether synaptic restoration translated into improved neuronal activity *in vivo*. Immunostaining for c-Fos revealed that clemastine treatment significantly increased the number of c-Fos^+^ cells in *Pdk1* icKO mice, indicating restored hippocampal neuronal activation (Fig. 5E and F).

Collectively, these findings demonstrate that clemastine not only rescues myelination deficits but also restores excitatory synaptic integrity and hippocampal neuronal activity in *Pdk1* icKO mice, underscoring its therapeutic potential for cognitive impairments associated with impaired myelination.

### Clemastine improves cognitive deficits in *Pdk1* icKO mice

**Fig. 6:**
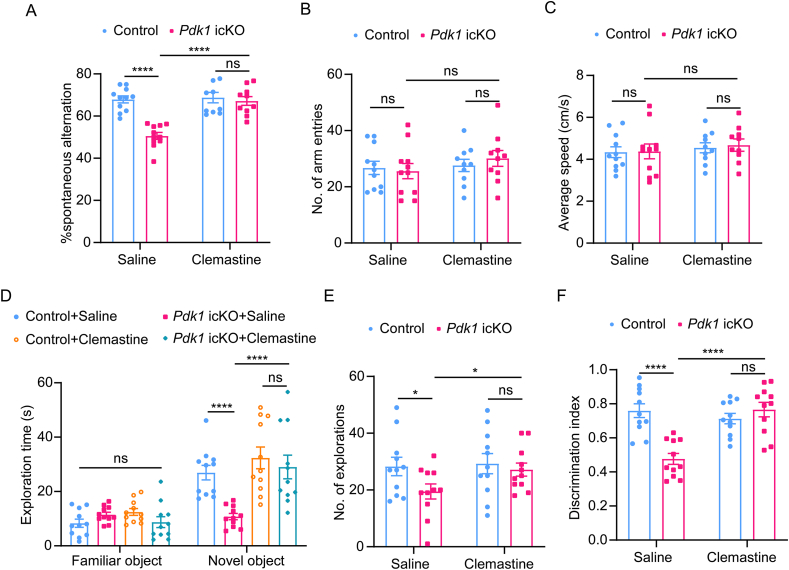
**Clemastine rescues memory deficits in *Pdk1* icKO mice** (A–C) Y-maze test. The Y-maze test showed that clemastine-treated *Pdk1* icKO mice exhibited significantly increased spontaneous alternation compared to saline-treated mutants (A). No significant differences were observed in the number of arm entries (B) or average speed (C) among groups, indicating unaffected locomotor activity. Data are presented as mean ± SEM (n = 10–11 per group; ∗∗∗∗, *p* < 0.0001; ns, not significant). (D–F) NOR test. While exploration time for the familiar object did not differ among groups, clemastine-treated *Pdk1* icKO mice spent significantly more time exploring the novel object compared to saline-treated mutants (D). Clemastine treatment significantly restored exploration events (E) and improved the discrimination index (F) in *Pdk1* icKO mice compared to saline-treated mutants. Data are presented as mean ± SEM (n = 10–11 per group; ∗, *p* < 0.05; ∗∗∗∗, *p* < 0.0001; ns, not significant).

Given that clemastine effectively rescued histological deficits in *Pdk1* icKO mice, we next assessed whether it could ameliorate associated cognitive impairments. Behavioral analyses were performed using the Y-maze and NOR tests in *Pdk1* icKO mice treated with clemastine from P21 to P58. In the Y-maze tests, spontaneous alternation rates were significantly increased in clemastine-treated *Pdk1* icKO mice compared to saline-treated mutants (Fig. 6A), while the total number of arm entries and average locomotor speed remained comparable between groups (Fig. 6B and C), indicating improved spatial working memory. Similarly, in the NOR test, clemastine-treated *Pdk1* icKO mice displayed increased exploration of the novel object, as reflected by both exploration time and exploration events, compared to saline-treated mutants (Fig. 6D and E). The discrimination index was also significantly elevated following clemastine administration (Fig. 6F), demonstrating enhanced recognition memory.

Collectively, these behavioral results provide compelling evidence that clemastine treatment effectively mitigates the cognitive deficits caused by oligodendroglial PDK1 deletion, consistent with the observed restoration of hippocampal myelination and excitatory synaptogenesis.

### Clemastine enhances Akt-mTOR signaling in *Pdk1* icKO mice

**Fig. 7:**
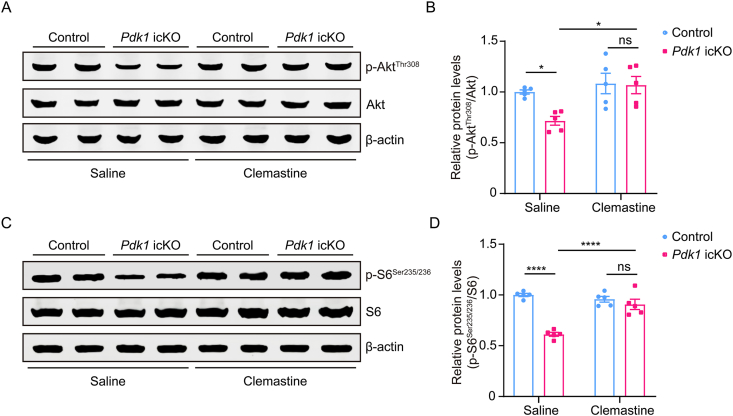
**Clemastine reactivates the Akt-mTORC1 pathway in *Pdk1* icKO mice** (A) Representative immunoblots of p-Akt^Thr308^ and total Akt in hippocampal lysates from control and *Pdk1* icKO mice treated with saline or clemastine. β-actin was used as a loading control. (B) Quantification of p-Akt^Thr308^ levels normalized to total Akt. Phosphorylation of Akt was significantly reduced in saline-treated *Pdk1* icKO mice compared to controls, and this decrease was rescued by clemastine treatment. Data are presented as mean ± SEM (n = 5 per group; ∗, *p* < 0.05; ns, not significant). (C) Western blotting analysis of p-S6^Ser235/236^ and total S6 in hippocampal lysates from the indicated groups. β-actin was used as a loading control. (D) Quantification of p-S6^Ser235/236^ levels normalized to total S6. A significant reduction in S6 phosphorylation was observed in saline-treated *Pdk1* icKO mice compared to controls. Clemastine treatment markedly restored p-S6^Ser235/236^ levels in *Pdk1* icKO mice. Data are presented as mean ± SEM (n = 5 per group; ∗∗∗∗, *p* < 0.0001; ns, not significant).

To investigate the molecular mechanisms by which PDK1 regulates hippocampal myelination, we examined the activity of the Akt-mTOR signaling pathway, a key downstream effector of PDK1that plays a critical role in oligodendrogenesis and myelination [43,44]. Akt activity was assessed by western blotting for phosphorylated Akt at threonine 308 (p-Akt^Thr308^) in hippocampal lysates. *Pdk1* icKO mice exhibited a significant reduction in the ratio of p-Akt^Thr308^ to total Akt compared to controls. Clemastine treatment substantially increased p-Akt^Thr308^ levels in *Pdk1* icKO mice (Fig. 7A and B), indicating restoration of Akt activity. To evaluate mTORC1 signaling, we examined the phosphorylation of ribosomal protein S6 at serine 235/236 (p-S6^Ser235/236^), a canonical readout of mTORC1 activity [45]. The relative levels of p-S6^Ser235/236^ to total S6 were significantly reduced in *Pdk1* icKO mice, whereas clemastine treatment markedly rescued these levels (Fig. 7C and D).

These results indicate that postnatal PDK1 ablation attenuates Akt-mTOR pathway activity in the hippocampus and that clemastine treatment effectively reactivates this signaling cascade, providing a potential mechanistic basis for its pro-myelination and cognitive rescue effects.

## Discussion

In the present study, we employed a *Pdk1* icKO mouse model to investigate the role of PDK1 in postnatal hippocampal myelination and cognitive development. Our findings demonstrate that postnatal ablation of PDK1 in OPCs impairs oligodendrogenesis and myelination, which is associated with deficits in excitatory synaptogenesis, neuronal activity, and cognitive performance. Notably, pharmacological intervention with clemastine effectively rescued both histological and behavioral abnormalities. Mechanistically, our findings suggest that clemastine restores myelination in *Pdk1* icKO mice, at least in part, by reactivating the Akt-mTOR signaling pathway.

Our findings extend our previous work using *Olig1-Cre*-mediated *Pdk1* cKO mice [22], which exhibited catastrophic myelination failure. While the constitutive model established the essentiality of PDK1 in OL development, it displayed severe spontaneous seizures starting at P14 and early lethality by P21. Such severe phenotypes precluded the investigation of long-term cognitive functions. By contrast, the inducible *NG2-CreERT2* strategy used here allowed temporal control of *Pdk1* deletion during a defined early postnatal window, bypassing the earliest waves of oligodendrogenesis and enabling survival into adulthood. This approach uncovered a previously inaccessible role for PDK1 in coordinating postnatal CNS myelination with synaptic transmission and cognitive functions.

Previous clinical studies have identified strong associations between *PDK1* mutations and ID [17,18]. While prior mouse models have established the essential role of PDK1 in neuronal development, including cortical migration, progenitor proliferation, and interneuron survival [[19], [20], [21]], the contribution of PDK1-dependent hypomyelination to cognitive impairment remained unexplored. Using an inducible and OPC-specific knockout strategy during early postnatal development, our results reveal that loss of PDK1 disrupts hippocampal myelination and leads to significant deficits in spatial working memory and recognition memory. The hippocampus, a key limbic structure underlying learning and memory [46], undergoes substantial postnatal myelination that is particularly vulnerable to genetic and environmental perturbations [[47], [48], [49]]. These findings suggest that PDK1-dependent hippocampal myelination may represent a potential mechanistic contributor to cognitive dysfunction associated with ID.

Notably, our results also reveal a distinct regional specificity in PDK1-dependent myelination. In addition to the hippocampus, significant hypomyelination was observed in the PFC [50,51], a critical hub for cognitive and sensory processing. This structural deficit aligns with the impaired spatial working memory and recognition memory observed in the Y-maze and NOR tests, which are primarily mediated by hippocampal-prefrontal circuits [29,30]. In contrast, cerebellar myelin integrity remained largely intact in *Pdk1* icKO mice. This lack of cerebellar involvement explains the intact general locomotor activity observed in our study.

Although myelin is classically recognized for facilitating rapid axonal conduction, emerging evidence indicates that it also plays a critical role in synaptogenesis and structural synaptic plasticity [39,42]. For example, developmental hypoxia models show concurrent impairments in myelination and synaptogenesis, which can be reversed by myelin restoration [39]. Consistent with these observations, we demonstrate that PDK1 deficiency in OPCs results in hippocampal hypomyelination, impaired excitatory synaptic structure, and reduced synaptic transmission, whereas pharmacological enhancement of myelination by clemastine restores synaptic structure. Importantly, in the context of such profound myelin deficiency, it is crucial to evaluate the structural integrity of the axons themselves. Ultrastructural analyses and NF200 staining indicate that axons remain structurally intact, suggesting that the observed cognitive and synaptic deficits primarily reflect functional consequences of impaired myelination and reduced oligodendroglial support rather than irreversible axonal loss. Collectively, these findings highlight the functional interplay between myelination and synaptogenesis as a potential therapeutic target for neurodevelopmental disorders.

Clemastine, an FDA-approved antihistamine with established clinical safety, has recently emerged as a potent promoter of OL differentiation and myelination [41,52,53]. Its pro-myelinating efficacy has been demonstrated across multiple neurological contexts, including: (i) aging mice, where clemastine promotes new myelin formation and alleviates age-related cognitive decline [42]; (ii) Alzheimer's disease models, where it enhances myelin renewal and improves cognitive performance [54]; and (iii) a Williams syndrome mouse model, where it promotes oligodendrogenesis and myelination, thereby ameliorating social behavioral deficits [55]. Our study extends these findings by demonstrating that clemastine mitigates myelination deficits and cognitive impairment induced by OPC-specific PDK1 deletion during the critical early postnatal period. The consistency of clemastine's effects across models underscores its robust promyelinating capacity and translational potential for treating myelination deficit-associated cognitive disorders.

Mechanistically, our BrdU birth-dating experiments further clarify that the myelination deficits in *Pdk1* icKO mice stem from an arrest in OPC differentiation rather than an impairment in their proliferative capacity. Although previous studies have suggested that PDK1 can influence cell proliferation and size in various contexts [56], our findings indicate that during the early postnatal hippocampal maturation window, PDK1 is specifically indispensable for the transition of OPCs into mature, myelinating OLs. PDK1 acts upstream of the Akt-mTOR pathway, which is crucial for oligodendrogenesis and myelin formation. Prior studies have shown that deletion of Akt in OPCs inhibits OL differentiation and CNS myelination [57], conditional mTOR knockout impairs OL differentiation and myelination [58], and loss of Raptor, an essential component of mTORC1, results in profound oligodendrogenesis deficits and severe hypomyelination [44]. Consistent with this, PDK1 deficiency reduced Akt and mTORC1 activity in the hippocampus, potentially underlying the observed impaired myelination phenotype. Clemastine treatment was associated with restoration of Akt and mTORC1 signaling in *Pdk1* icKO mice, providing a plausible mechanistic basis for its observed therapeutic effects. Interestingly, clemastine has been reported to exert pro-myelination effects through antagonism of muscarinic receptor 1 (M1R) [59,60], raising the possibility of crosstalk between M1R signaling and Akt-mTOR pathway regulation in oligodendroglial cells, which warrants further investigation.

In summary, our study identifies a critical role for PDK1-dependent postnatal hippocampal myelination in cognitive development and provides novel mechanistic insights into myelination deficit-associated ID. Importantly, clemastine administration ameliorates both myelination deficits and cognitive impairments caused by oligodendroglial PDK1 ablation, supporting its potential as a translational therapeutic strategy for neurodevelopmental disorders involving impaired myelination.

## Author contributions

G.C., Y.H., J.L., and J.C. contributed to the conception of the study. P.Qiao., H.W., P.Qu., L.G., Y.Z., T.Z., and J.C. performed experiments and analyzed the data. G.C., H.W., P.Q., and J.C. wrote the manuscript with inputs from all authors.

## Data availability

The data that support the findings of this study are available within the article and its supplementary materials.

## Declaration of competing interests

The authors declare that they have no known competing financial interests or personal relationships that could have appeared to influence the work reported in this paper.

## Figures and Tables

**figs1:**
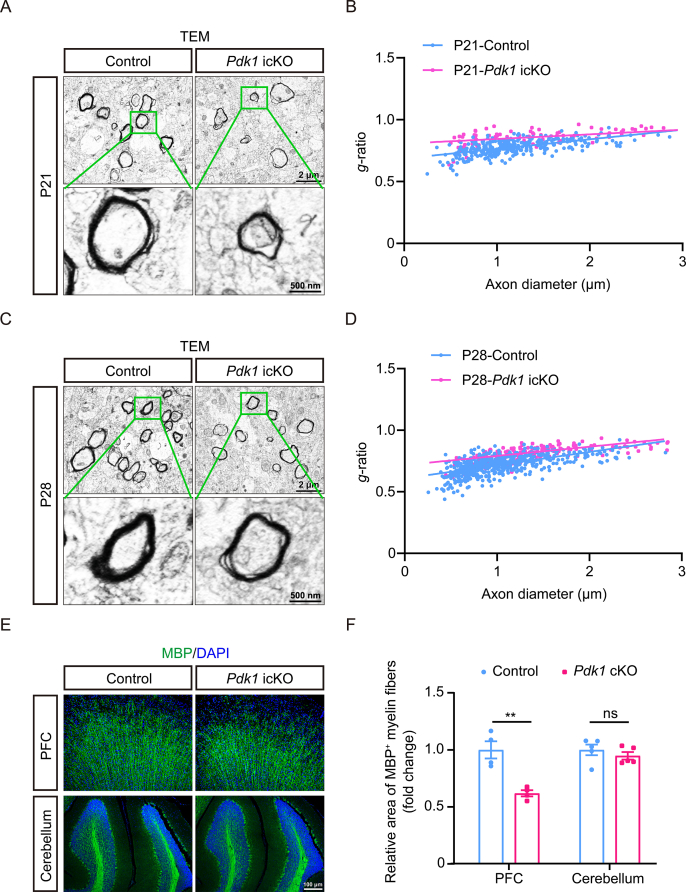


**figs2:**
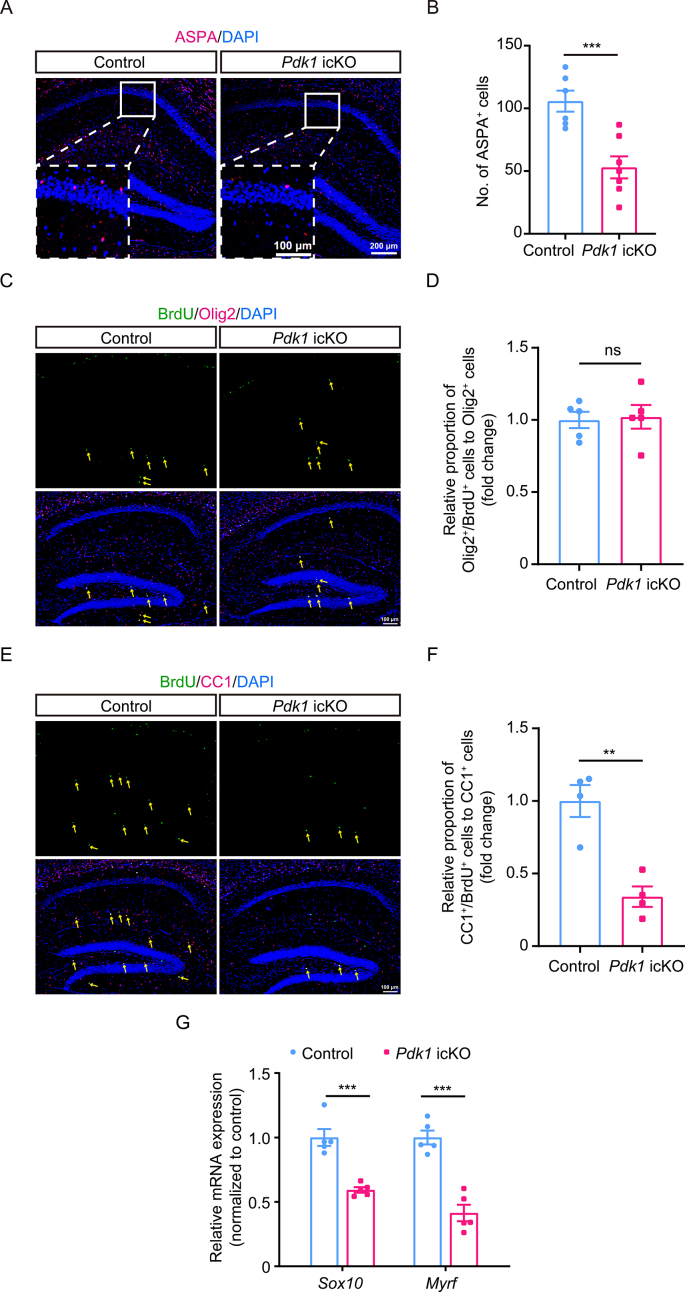


**figs3:**
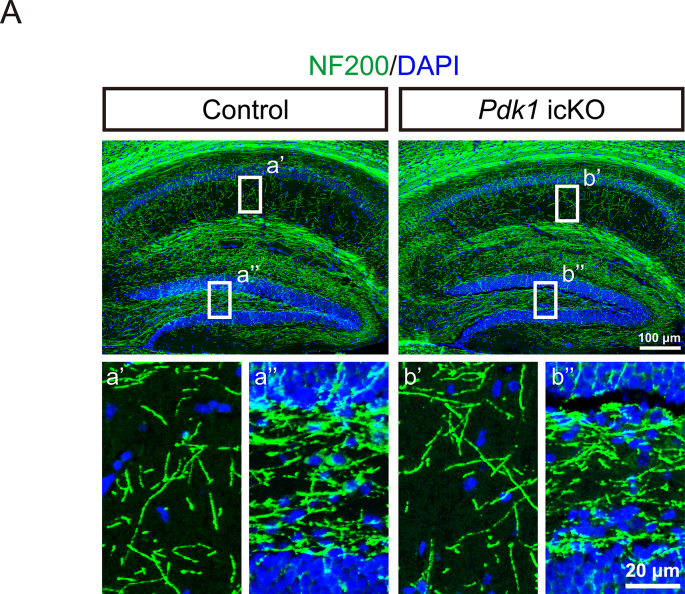


**figs4:**