# Supplementary material for: Targeting enhancing myelin regeneration reverses cognitive deficits in a mouse model of intellectual disability
Source: Neurotherapeutics. 2026 Apr 13;23(3):e00906. doi: 10.1016/j.neurot.2026.e00906 (PMC13092863; doi:10.1016/j.neurot.2026.e00906)
Supplement: Multimedia component 1 [file mmc1.docx]

**Supplementary Information**

**Title:** Targeting Enhancing Myelin Regeneration Reverses Cognitive Deficits in a Mouse Model of Intellectual Disability

**Authors:** Pingping Qiao**^*^**^,#^, He Wang**^†^**^,#^, Pingping Qu**^‡^**, Lifang Guo**^§^**, Yanbo Zhou**^†^**, Tingting Zeng**^†^**, Jiang Chen**^¶,||^**, Jian Li**^‡^**^,^**^||^**, Yimin Hu**^**^**^,^**^||^**, Guiquan Chen**^*^**^,^**^||^**

**^||^ Corresponding authors:**

chenguiquan@nju.edu.cn (G.C.); guyueym@pumcderm.cams.cn (Y.H.); lijian@njjlzy.com (J.L.); and chenjiang1njdth@163.com (J.C).

**List of Supplementary Information:**

Fig. S1-S4

Table S1-S4


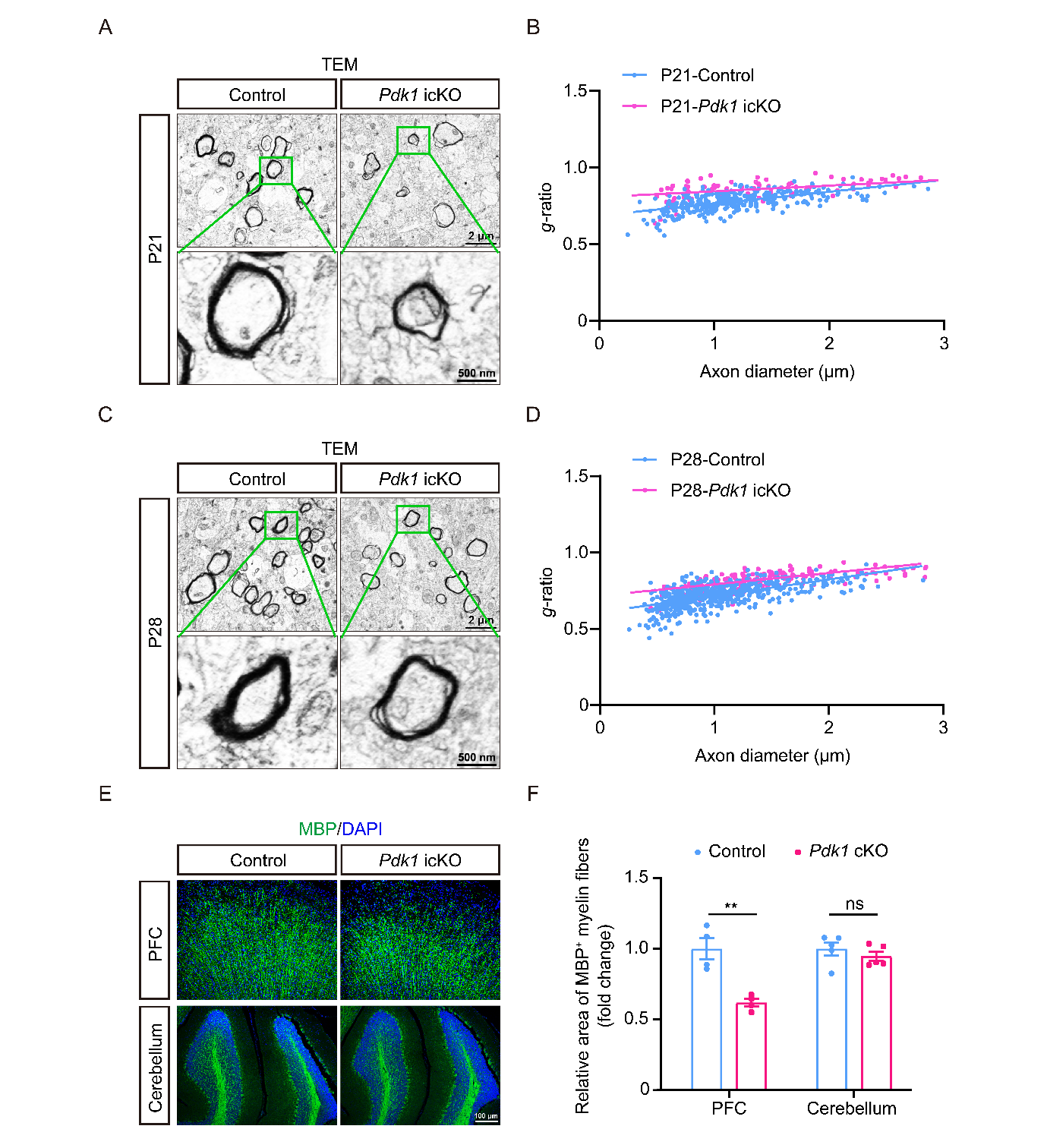


**Fig. S1 Postnatal loss of PDK1 leads to regional hypomyelination and reduced myelin thickness in the CNS**

(A) Representative TEM images of myelinated axons in the hippocampus at P21. Lower panels show high-magnification views of the boxed areas in the upper panels, highlighting thinner myelin sheaths in control and *Pdk1* icKO mice. Scale bars: 2 μm (upper) and 500 nm (lower). (B) Scatter plot of *g*-ratio values as a function of axonal diameter for individual axons at P21. The higher regression line for *Pdk1* icKO mice (pink) compared to controls (blue) indicates a thinner myelin sheath across various axon sizes. (Control: *g*-ratio = 0.77896741; *Pdk1* icKO: *g*-ratio = 0.858915). Data are presented as mean ± SEM (n = 3 mice per genotype). (C) Representative TEM images of myelinated axons at P28, showing persistent hypomyelination in *Pdk1* icKO mice. Scale bars: 2 μm (upper) and 500 nm (lower). (D) Scatter plot of *g*-ratio values relative to axonal diameter at P28. Consistent with P21, the *g*-ratio is significantly increased in *Pdk1* icKO mice, indicating sustained impairments in myelin thickness. (Control: *g*-ratio = 0.72125; *Pdk1* icKO: *g*-ratio = 0.808295). Data are presented as mean ± SEM (n = 3 mice per genotype). (E, F) Representative MBP (green) immunofluorescence images (E) and quantitative analysis (F) of myelination in PFC and cerebellum of control and *Pdk1* icKO mice at P60. Nuclei are counterstained with DAPI (blue). MBP immunoreactivity was significantly reduced in the PFC of *Pdk1* icKO mice compared with controls, whereas no significant difference in MBP staining was observed in the cerebellum. Data were shown as mean ± SEM (n = 4-6 per genotype; ***, *p*<0.001; ns, not significant). Scale bars: 100 μm.


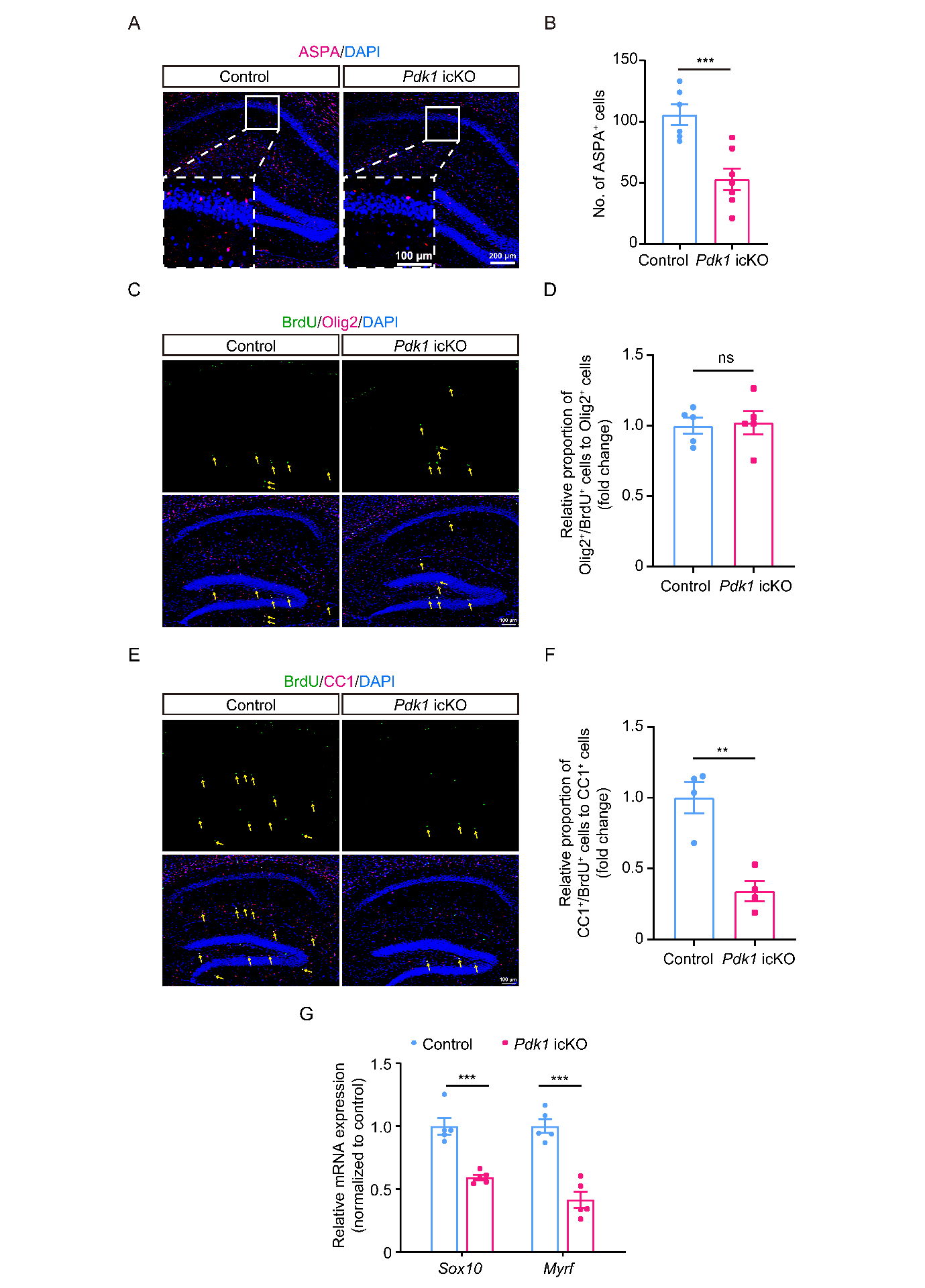


**Fig. S2 Postnatal PDK1 deletion impairs OPC differentiation without affecting proliferation**

(A) IHC staining for ASPA (red) in brain sections of control and *Pdk1* icKO mice. Nuclei were counterstained with DAPI (blue). Scale bars: 200 μm (overview), 100 μm (magnified views). (B) Quantification of ASPA^+^ cells revealed a significant reduction in *Pdk1* icKO mice compared to controls. Data are presented as mean ± SEM (n = 6-7 per genotype; ***, *p*<0.001). (C) Representative IHC images of BrdU (green), Olig2 (red), and DAPI (blue) staining in the hippocampus of control and *Pdk1* icKO mice 2 hours after a single BrdU pulse at P13. Arrows indicate BrdU⁺/Olig2⁺ cells. Scale bar, 100 μm. (D) Quantification of the relative proportion of BrdU⁺ cells among Olig2⁺ oligodendroglial lineage cells. No significant difference was observed between control and *Pdk1* icKO mice. Data were shown as mean ± SEM (n=5 per genotype; ns, not significant). (E) Representative images of BrdU (green), CC1 (red), and DAPI (blue) staining in the hippocampus of control and *Pdk1* icKO mice following BrdU pulse labeling (P13-P15) and analysis at P28. Arrows indicate BrdU⁺/CC1⁺ differentiated OLs. Scale bar, 100 μm. (F) Quantification of the relative proportion of BrdU⁺ cells among CC1⁺ mature OLs. The percentage of newly generated differentiated OLs was significantly reduced in *Pdk1* icKO mice compared with controls. Data were shown as mean ± SEM (n = 4 per genotype; ns, not significant). (G) qRT-PCR analysis showing relative mRNA expression levels of *Sox10* and *Myrf* in the hippocampus of control and *Pdk1* icKO mice. Expression levels were normalized to control samples. Data are presented as mean ± SEM (n = 5 per genotype; ***, *p* < 0.001).


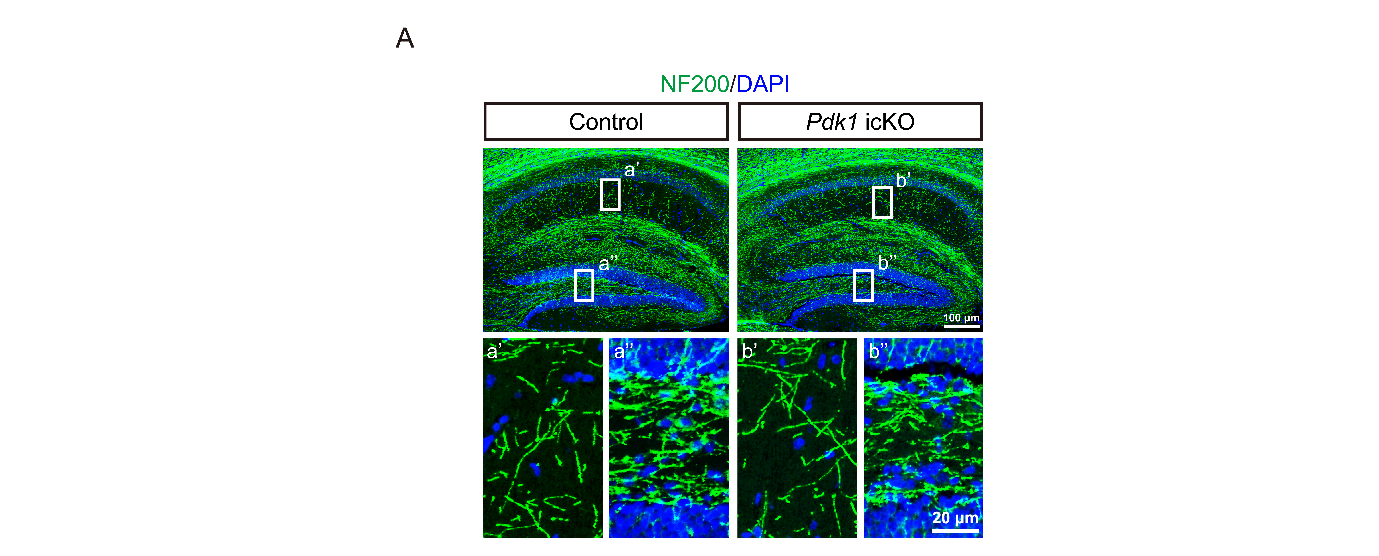


**Fig. S3 Preservation of axonal structural integrity in the hippocampus of *Pdk1* icKO mice**

(A) Representative IHC images of NF200 (green) and DAPI (blue) in the hippocampus of control and *Pdk1* icKO mice. No typical features of axonal degeneration, including axonal fragmentation, swelling, or beading of NF200⁺ signals, were detected. Scale bars: 100 μm (low magnification) and 20 μm (high magnification).


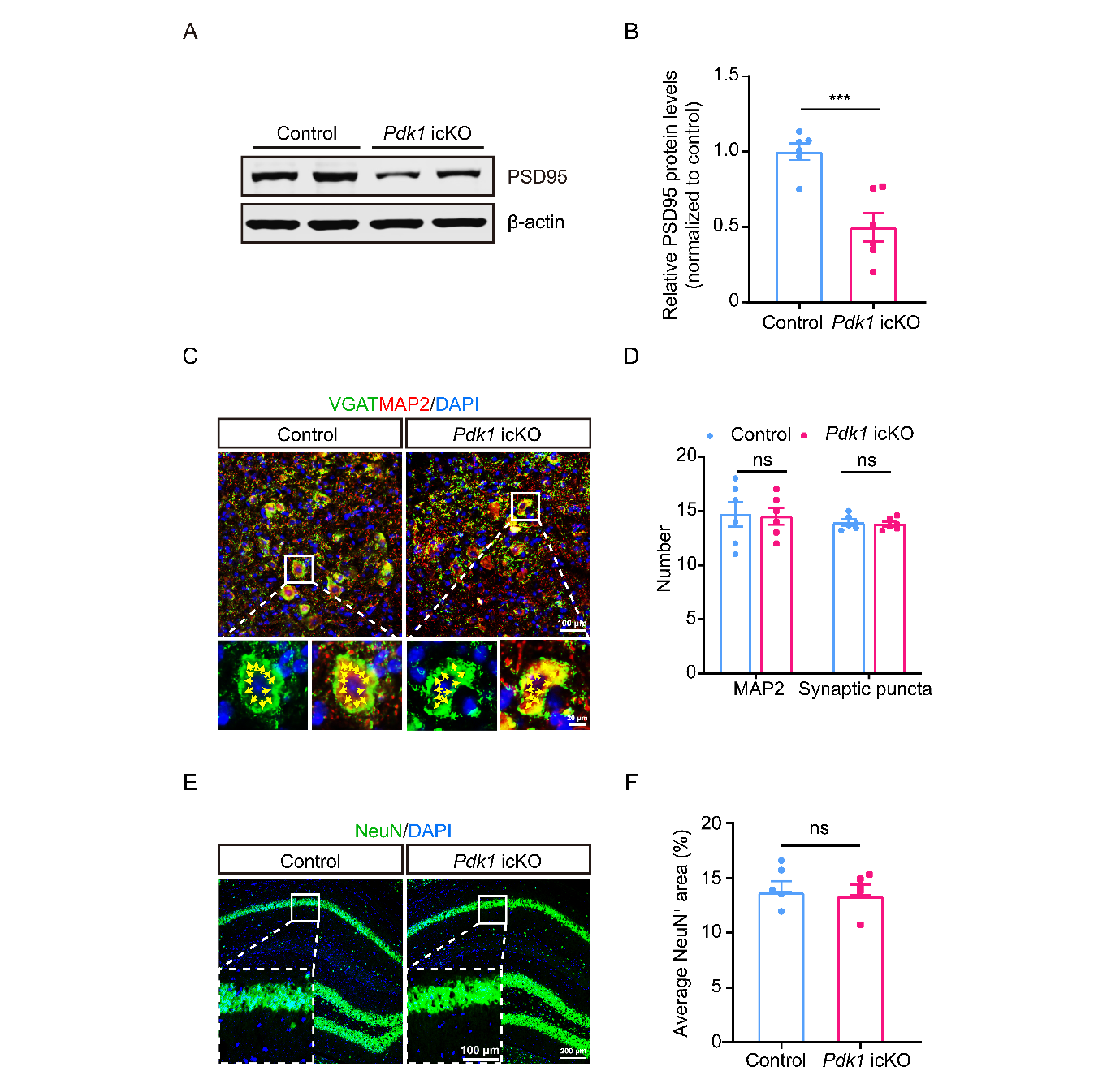


**Fig. S4 PDK1 deletion selectively impairs excitatory synaptic proteins without affecting inhibitory synapses or neuronal density**

(A) Representative Western blot analysis of PSD95 protein levels in hippocampal lysates from control and *Pdk1* icKO mice. β-actin was used as a loading control. (B) Quantification of PSD95 protein levels normalized to control. PSD95 expression was significantly reduced in *Pdk1* icKO mice. Data are presented as mean ± SEM (n = 6 per genotype; ***, *p* < 0.001). (C) Representative images of co-immunostaining for MAP2 (red) and VGAT (green) in brain sections from *Pdk1* icKO and control mice. Nuclei are counterstained with DAPI (blue). Scale bars: 100 μm (overview), 20 μm (magnified views). (D) Quantification showed no significant difference in the density of VGAT^+^ puncta around MAP2^+^ neurons between genotypes. Data are presented as mean ± SEM (n = 6 per genotype; ns, not significant). (E, F) IHC for NeuN (green) in brain sections of *Pdk1* icKO and control mice. Nuclei were counterstained with DAPI (blue). No significant difference was detected in the NeuN^+^ area between genotypes. Data are presented as mean ± SEM (n = 5 per genotype; ns, not significant). Scale bar: 200 μm (low magnification images), 100 μm (high magnification images).

**Table S1.**

List of antibodies.

| Antibodies | Source | Identifier |
| --- | --- | --- |
| Rabbit monoclonal anti-PDK1 | Abcam | Cat# ab52893, RRID: AB_881962 |
| Rabbit polyclonal anti-β-actin | GenTex | Cat# CTX124212, RRID: N/A |
| Rat monoclonal anti-MBP | Millipore | Cat# MAB386, RRID: AB_94975 |
| Mouse monoclonal anti-Olig2 | Millipore | Cat# MABN50, RRID: AB_10807410 |
| Rabbit monoclonal anti-PDGFRα | Cell Signaling Technology | Cat# 3174, RRID: AB_2162345 |
| Mouse monoclonal anti-CC1 | Calbiochem | Cat# op-80, RRID: AB_2057371 |
| Rabbit polyclonal anti-ASPA | Millipore | Cat# ABN1698, RsRID: AB_2827931 |
| Rabbit polyclonal anti-NeuN | Millipore | Cat# ABN78, RRID: AB_10807945 |
| Mouse monoclonal anti-GFAP | Santa Cruz | Cat# sc-65,343, RRID: AB_783553 |
| Rabbit polyclonal anti-VGlut1 | Synaptic Systems | Cat# 135 303, RRID: AB_887875 |
| Rabbit polyclonal anti-VGAT | Synaptic Systems | Cat# 131 003, RRID: AB_887869 |
| Mouse monoclonal anti-MAP2 | Millipore | Cat# MAB3418, RRID: AB_94856 |
| Rabbit polyclonal anti-Homer1 | Synaptic Systems | Cat# 160 003, RRID: AB_887730 |
| Rabbit polyclonal anti-PSD95 | Abcam | Cat# ab18258, RRID: AB_444362 |
| Guinea pig polyclonal anti-c-Fos | Oasis | Cat# OB-PGP080, RRID: AB_2941873 |
| Rabbit polyclonal anti-Phospho-S6 Ribosomal Protein (Ser235/236) | Cell Signaling Technology | Cat# 2211, RRID: AB_331679 |
| Rabbit polyclonal anti-S6 | Abclonal | Cat# A6058, RRID: AB_2766731 |
| Rabbit monoclonal anti-Akt | Cell Signaling Technology | Cat# 4691, RRID: AB_915783 |
| Rabbit monoclonal anti-Phospho-Akt (Thr308) | Cell Signaling Technology | Cat# 13038, RRID: AB_2629447 |
| Goat Anti-Rabbit IgG, IRDye® 800CW Conjugated antibody | LI-COR Biosciences | Cat# 926-32211, RRID: AB_621843 |
| Goat Anti-Mouse IgG, IRDye® 800CW Conjugated antibody | LI-COR Biosciences | Cat# 926-32210, RRID: AB_621842 |
| Alexa Fluor® 488 AffiniPure® Goat Anti-Rabbit IgG (H +L) | Jackson ImmunoResearch Labs | Cat# 111–545-003, RRID: AB_2338046 |
| Alexa Fluor® 594 AffiniPure® Goat Anti-Mouse IgG (H+L) | Jackson ImmunoResearch Labs | Cat# 115-585-003, RRID: AB_2338871 |
| Alexa Fluor® 488 AffiniPure® Donkey Anti-Guinea Pig IgG (H+L) | Jackson ImmunoResearch Labs | Cat# 706-545-148, RRID: AB_2340472 |
| Alexa Fluor® 488 AffiniPure® Donkey Anti-Rat IgG (H+L) | Jackson ImmunoResearch Labs | Cat# 712-545-150, RRID: AB_2340683 |

**Table S2.**

List of chemicals and recombinant proteins.

| Name | Source | Identifier |
| --- | --- | --- |
| DAPI | Sigma-Aldrich | Cat# D9542 |
| TrueGold myelin kit | Oasis | Cat# BK-AC001 |
| RNAiso Plus | Takara | Cat# 9109 |
| Tamoxifen | Sigma-Aldrich | Cat# H6278 |
| BrdU | Sigma-Aldrich | Cat# B5002 |
| Clemastine | Selleck | Cat# S1847 |
| TUNEL BrightGreen Apoptosis Detection Kit | Vazyme | Cat# A112-03 |
| ChamQ SYBR qPCR Master Mix | Vazyme | Cat# Q311-02 |
| HiScript II Q RT SuperMix for qPCR (+gDNA wiper) | Vazyme | Cat# R223-01 |
| Picrotoxin | Tocris Bioscience | Cat# 1128 |
| [Bicuculline](https://www.absin.cn/bicuculline/abs812832.html) | Absin | Cat# abs812832 |

**Table S3.**

List of primers for qRT-PCR.

| Name | Primer Sequence 5’-3’ | Identifier |
| --- | --- | --- |
| *Pdk1*-F | CCTTCAGGAGTTGCTTGATTTT | N/A |
| *Pdk1-*R | ACATTTTGGCTGGTGACAGG | N/A |
| *Sox10*-F | CAGTACCCTCACCTCCACAA | N/A |
| *Sox10*-R | CGCCGAGGTTGGTACTTGTA | N/A |
| *Myrf*-F | TCTAACCCCAAGCACTCAGG | N/A |
| *Myrf*-R | GTTCTTGGTCTTGCTCTGCC | N/A |
| *Gapdh*-F | GAGTGTTTCCTCGTCCCGT | N/A |
| *Gapdh*-R | ACAATCTCCACTTTGCCACTG | N/A |

**Table S4.**

List of software and algorithms.

| Name | Primer Sequence 5’-3’ | Identifier |
| --- | --- | --- |
| Leica confocal software | http://softadvice.informer.com/Leica_Confocal_Software.html | N/A |
| SS-MCS Microscopic Confocal Scanning System | http://www.ss-raman.com/pd.jsp?id=50 | N/A |
| Olympus camera software | https://www.olympuslifescience.com/en/support/downloads/ | N/A |
| LI-COR Image Studio | http://www.dxy.cn/bbs/topic/34994594 | N/A |
| GraphPad Prism 8 | https://www.graphpad.com/scientificsoftware/prism/ | N/A |
| Adobe Photoshop CS6 | https://creative.adobe.com/products/dow | N/A |
| Adobe Illustrator | https://creative.adobe.com/products/dow | N/A |
| Image J | https://imagej.nih.gov/ij/download.html | N/A |
